# Comparative analysis for producing sweetpotato pre-basic seed using sandponics and conventional systems

**DOI:** 10.1080/15427528.2019.1674758

**Published:** 2019-10-12

**Authors:** Phabian Makokha, Reuben T. Ssali, Srinivasulu Rajendran, Bramwel W. Wanjala, Lexa G. Matasyoh, Oliver K. Kiplagat, Margaret A. McEwan, Jan W. Low

**Affiliations:** a International Potato Center, Nairobi, Sub-Saharan Africa, Kenya; b International Potato Center, Kumasi, Sub-Saharan Africa, Ghana; c Kenya Agricultural and Livestock Research Organization, National Agricultural Research Laboratories, Nairobi, Kenya; d Department of Biological Sciences, University of Eldoret, Eldoret, Kenya; e Department of Biotechnology, University of Eldoret, Eldoret, Kenya

**Keywords:** Cost effectiveness, potassium, sandponics, screenhouse substrates, sweetpotato pre-basic seed

## Abstract

In Sub-Saharan Africa, sweetpotato pre-basic seed is multiplied in screenhouses using a sterilized soil substrate. This is expensive and unsustainable. The use of sand substrate with a fertigation system (“sandponics”), is an alternative. The study compared the cost-effectiveness for pre-basic seed production using the sandponics system to the conventional soil substrate for four genotypes. A randomized complete block split plot design was used, and data collected on vine traits over six harvests. Real-time cost data were collected for cost-effectiveness analysis. Results showed a highly significant (p < .0001) 21.8% increase in the vine multiplication rate under the sandponics system. The cost of producing one sweetpotato node in sandponics was significantly lower by 0.009 US$. The cost-effectiveness of producing pre-basic seed in sandponics varied among the genotypes. The future use of sandponics is discussed with respect to the availability of soluble inorganic fertilizers, varietal specific response to nutrients, and labor implications.

## Introduction

Sweetpotato (*Ipomoea batatas* (L.) Lam) is an important crop in Sub-Saharan Africa (SSA). It plays a critical role as an income and food security crop for many households (Amajor et al. 2014; Byju and George 2005; Lebot 2008; Motsa et al., 2015). Other advantages of sweetpotato are: flexible planting dates, a short maturity period (3–4 months), suitable for distribution as part of post conflict and disaster relief programs, it requires minimum inputs and can be grown on infertile soils where grain crops may fail, once established it is drought tolerant and many varieties have potential for piecemeal harvesting over an extended period of time (Ndolo et al. 2001). The crop has a high root yield potential of 20–50 t ha^−1^ (Kivuva et al. 2014). However, this yield potential is not realized in SSA, where productivity is less than 10 t ha^−1^ (FAOSTAT 2017). Assessment of sweetpotato production constraints in SSA shows that limited access to disease-free planting materials and improved varieties are the major factors contributing to low yields (Andrade et al. 2009; Gibson et al. 2009; Gibson, Namanda, and Sindi 2011b). These findings underline that a sustainable seed system is vital in improving sweetpotato productivity in SSA, as has been demonstrated in Shandong province, China (Fuglie et al. 1999). A functional sweetpotato seed system should provide timely and affordable access for different types of farmers to adequate quantities of quality planting material of preferred varieties (Barker et al. 2009). However, in SSA, a major bottleneck in the sweetpotato seed system has been the availability of sufficient quantities of pre-basic seed to supply commercial sweetpotato seed producers. Currently, the dominant practice is for pre-basic seed to be multiplied in screenhouses in pots or boxes using a sterilized soil substrate. The use of sterilized soil is expensive, unsustainable, and may not achieve optimal vine multiplication rates (VMR) .

In the past, methyl bromate was used to sterilize soil; however, this has been banned and the alternative of sterilizing soil using steam is very costly. The use of sand substrate with a fertigation system, also referred to as “sandponics” has been proposed as an alternative to the conventional soil, manure, gravel substrate mix (“conventional soil substrate type”) used in screenhouse production. Previous work has optimized the nutrient media for sweetpotato vine multiplication using the sandponics system (Makokha et al. 2018). However, the cost-effectiveness of using the sandponics system compared to the conventional soil substrate method has not been established. Wanjala, B.W., Rajendran, S., Makokha, P., Ssali, R.T., McEwan, M., Kreuze, J.F., and Low, J.W. (2019, unpublished) found that although the use of a sandponics system and trellising increased the VMR by 33%, use of the conventional soil substrate was still more cost-effective. However, that experiment was based on the results for two vine harvests and therefore the optimal production period may not have been reached. Therefore, this study was conducted to compare the use of a sandponics system with an optimized nutrient media formula with the conventional soil substrate to establish which method was more cost-effective.

## Materials and methods

The study was conducted between June 2018 and March 2019 at the Kenya Plant Health Inspectorate Service – Plant Quarantine and Biosecurity Station (KEPHIS – PQBS), Muguga, Kenya, located at 1° 11ʹ 0” South, 36° 39ʹ 0” East and an altitude of 1,950 m asl. Two types of substrates for rapid multiplication of sweetpotato pre-basic seed in screenhouses were evaluated to measure their cost-effectiveness. The first type used sterilized sand with a fertigation system also known as “sandponics” and the second used the conventional method of sterilized soil. Sand substrate was sterilized by soaking in 10% sodium hypochlorite (NaOCl) for 10 min, rinsed three to four times with tap running water to remove NaOCl residues (Otazu 2010; Mbiri et al. 2015), and put on a raised rack to dry prior to potting. The conventional soil media was composed of forest top soil, cow manure, and gravel in the ratio of 5:2:1. This was sterilized by steaming in a steam boiler for 30 min at 82°C using diesel as the source of energy.

Samples of irrigation water, sterilized sand, and conventional soil substrates were taken for analysis by Crop Nutrition Laboratories, Nairobi, Kenya before the experiment started. The pH of the sand substrate was 6.8, with nutrient elements below detectable levels. The chemical and physical properties of the soil profile were documented (see Supplementary Table S1).

The laboratory water analysis report (Table S2) was used to adjust the nutrient concentrations in the sweetpotato vine multiplication media and optimal nutrient media used in sandponics system worked out from fertilizer formulations (Table S3). Optimization of sandponics system nutrient media was limited to N, P, Ca, S, and B which are the key elements reported to favor sweetpotato vine growth (Taraken et al., 2010; Makokha et al. 2018) and potassium was deliberately omitted given that its role in the growth of sweetpotato comes in later, at least 7 weeks after planting (Bourke 1985; Taraken et al. 2010). The pH of the final solution was adjusted to 5.8 using HI98107 pH meter (Hanna Instruments Ltd, UK) by adding 5 mL of 0.1 M phosphoric acid.

The experiment was subjected to a randomized complete block split plot design (RCBSPD) with repeated measures. Two substrate types were used for the study, i.e. sand substrate and conventional soil media. Each treatment combination (substrate type × variety) was replicated five times in four blocks. The substrate type was a whole plot factor while each variety was a sub-plot factor. Genotypes Irene, Kabode, Ejumula, and Gweri were used in the study selected based on their growth morphology as erect, semi-erect, spreading and extremely spreading, respectively (Huamán 1991; Tumwegamire et al. 2014).

Plastic pots measuring 18 cm diameter and 20 cm slanting height were filled with 5.5 kg and 4.0 kg of sterilized sand or sterilized soil media, respectively. Planting material of the four sweetpotato genotypes were obtained by taking three-node cuttings from hardened pathogen-tested plants maintained at KEPHIS-PQBS, Muguga, Kenya. Prior to planting, pots were irrigated and fertigated in the conventional soil substrate method and sandponics system, respectively, to moisten the substrates and avoid injuring cuttings during planting. Irrigation water and fertigation nutrient media was supplied from elevated tanks connected to drip lines in the sandponics system and conventional soil substrate. Manual valves allowed the distribution of water and nutrients by gravity through surface pipes and drippers.

Ten plants were planted per pot approximately 3 cm apart from each other with two nodes buried in the substrate and one node above the surface. Three grams of diammonium phosphate (18:46:0) was applied per pot at planting in the conventional soil substrate. Subsequent irrigation (conventional soil substrate) and fertigation (sandponics system) were guided by Irrometer SR 12” (Irrometer Company Inc., CA, USA). Leaching of nutrients following two fertigations was done as described by (Chen 2013) to avoid accumulation of salts to toxic levels in the sandponics system. Heat controllers, sensors and cooling air extract fans maintained a mean temperature range of 26 ± 4°C which is optimal for sweetpotato vine growth (Chen 2013) and this was monitored by HOBO U12-013 data logger (Onset Computer Corp., Bourne, MA, USA). For the convential soil substrate method, calcium ammonium nitrate (27% N) fertilizer was applied 2 weeks after each harvest at the rate of 3 g per pot.

The vines were harvested six times at 42-day intervals, therefore, over the 9-month crop calendar, one vine harvest and five ratoons (subsequent grow outs) were carried out. The number of harvests was based on findings from earlier studies using a trellising technique and sandponics system (Wanjala et al. 2019, unpublished) where after two harvests the VMR had increased by 33% in the sandponics system compared to the conventional soil substrate method, but the latter conventional soil substrate was more cost-effective. At harvest, 20–30 cm cuttings with at least three nodes were harvested starting from the vine tip and leaving two nodes above the surface for regrowth. At 42, 84, 126 and 210 days after planting (DAP) plants for all four genotypes in the two distinct systems were randomly sampled. From each sampled plant, leaves were selected from the 7^th^ to 9^th^ open leaf blades from the shoot tip, and samples from the same genotype and substrate system bulked as one composite sample for anlaysis (O’Sullivan, Asher, and Blamey 1997). Analysis was conducted for key nutrients for deficiency, toxicity and for recommended range of concentration sampled. The leaf tissue analysis was done at Crop Nutrition Laboratories, Nairobi, Kenya.

### Data collection

The data collection tools in the experiment were used between June 2018 and March 2019 to capture key agronomic and production cost variables for screenhouse production of sweetpotato pre-basic seed using the two different substrates systems.

The following data on sweetpotato vine morphological and yield traits were collected. Average leaf area was determined by measuring leaf length (L) and width (W) at the widest part of the fifth leaf from the tip of the main stem and the product L × W was used to compute for leaf area (cm^2^/plant). Average petiole length was deduced by measuring the point between leaf attachment to the main stem and the leaf, and the average vine internode length. The measurements for leaf area, petiole and vine internode length were done on the fifth leaf from the tip of the main stem. Data on average vine length were determined by measuring the main stem length from the surface of the substrate in the pot to the tip. All the aforementioned measurements were done using a meter ruler on 50% plant population per pot at each harvest.

Also at harvest, the total number of nodes on all vines per pot were counted and recorded. Vine multiplication rates in the two different substrate methods were calculated by dividing the total number of nodes produced by three (a three-node cutting constitutes a unit of planting material) which is the ideal “seed” for sweetpotato. Harvested vines from each pot were also weighed using an electronic digital LCD scale SF–400 (5 kg) and total fresh weight determined.

For the cost data, the study hypothesized that in a respresentative production period, the production cost per node in the sandponics system would be lower than in the conventional soil substrate method. The hypothesis was tested using cost-effectiveness or least-cost combination method. This method is part of constant effect method and is normally used in low-income settings to deal with intangible benefits. The intangible benefits were determined on a present worth basis and the least expensive alternative combination of tangible costs that would realize the same intangible benefits (Gittinger 1985). The cost-effectiveness analysis was used to identify the most cost-effective substrate method to produce sweetpotato pre-basic seed. Since the study was based on experimental basis, the study collected cost data in the sandponics system and conventional soil substrate vine propagation methods based on real-time basis.

The production cost calculation in the experiment was carried out over a crop calendar period of 9 months and hence the cost estimates were restricted to activities carried out during this period. The following procedure was used to gather information on costs for the two different substrate methods; (i) selection of team members who were directly involved in the production activities, (ii) preparation of the crop calendar, (iii) mapping out operational activities and inputs, and (iv) mapping out costs information and share of allocation of inputs. Data collection questionnaires were prepared as micro-log and macro-log sheets. The input cost template and labor cost template micro-log sheets (Table S4 and S5, respectively) are daily record sheets which monitor daily amounts of input usage and activities in the experiment for each laborer in that order. After data were recorded in the micro-log sheet for each laborer, the information was then transferred to the input template and labor cost template macro-log sheets (Table S6 and S7, respectively) and later transferred to the cost calculation sheet developed in MS office excel program (Table S8). The data for each production activity in the sandponics system and conventional soil substrate method were collected and reported separately using the micro and macro-log sheets.

The costs were classified into variable and fixed costs. Variable costs included labor, inputs and consumable costs. The cost of labor was estimated based on the daily wage rate and the number of man-days used by each laborer for each production activity. The input costs were calculated using data on the quantity and prices of inputs used. Finally, the costs for consumables were also included as part of variable costs. Fixed costs are defined as those costs that occur regardless of quantity produced. The team identified the types of equipment that were used for producing sweetpotato pre-basic seed during the experimental period. Once each type of equipment was identified, the team identified the life (years) of the equipment and the fixed cost was estimated by adding-up depreciation, interest on average investment and insurance and taxes. Summing both variable and fixed costs, the total cost of production was then estimated. In addition to total production costs, 10% overhead was included for both substrate methods to take into account costs for water and electricity, which were paid directly by the government institution. The study also monitored pre and post-harvest losses which were then accounted for in the total costs. The study estimated the cost of producing sterilized sand and soil substrate separately and included these into the cost calculations for the sandponics system and conventional soil substrate, respectively. The detailed fixed, variable costs and consumables used to calculate the total cost of production in the two substrate methods are as shown in Table S8. The cost of producing sterilized sand is shown in Table S9. The cost of sand per kilogram is inclusive of transport cost. Since sand was readily available close by, the cost of sand per kilogram is relatively lower. The sterilized soil was bought from KEPHIS-PQBS, Muguga, Kenya, so, this price was used in the cost estimation (i.e., US$ 0.20 or KSH 20.0 per kg). The exchange rate for converting Kenyan shillings (KSH) into US dollar (US$) was 1 US$ equivalent to 100 KSH.

### Data analysis

Statistical analyzes for the agronomic data on vine yield and traits were conducted using SAS 9.4 version (SAS Institute Inc., 2013) and Stata version 14.1 (STATA 14.1 version, 2015). Data for the two substrate methods were analyzed using the proc t-test to compare vine productivity in the sandponics system and the conventional soil substrate method. The effects of treatments on varietal response and their interactions were evaluated at p ≤ 0.05 using the general linear model procedure.

Statistical model;

$$Y(ij)=f^{\prime }\;(W_{i},S_{ij})\beta +\gamma_{i}+E_{ij}$$

where W = whole plot factor, S = sub plot factor, ᵞ = whole plot effect, E = random error, i = block effect, j = runs within the block and ij = j^th^ response in the i^th^ block.

Node data for vine production were fitted into a model:

$$\begin{array}{rl} & Y\left(ijkl\right)=var_{i}+sub_{j}+REP_{k}+{\left(var\times sub\right)}_{ij}+{\left(var\times REP\right)}_{ik}\\ & \;\;\;\;\;\;\;\;\;\;\;\;\;\;\;\;\;\;\;\;\;\;\;+{\left(sub\times REP\right)}_{jk}+Ei_{jkl} \end{array}$$

where Y = the number of nodes, var = sweetpotato varieties, sub = substrate (sandponics system and conventional soil substrate method), REP = replication (1,2,3,4,5), E = errors generated in repeated measures. Among them, effects written by lower case letters are fixed effects, and effects written by upper case letters are random effects.

A detailed cost-effectiveness analysis was conducted for the two methods of sweetpotato vine production based on all relevant indicators as described by Mateus-Rodriguez et al. (2013). Total production cost (TPC, Kenyan Shillings (KSH) per crop calendar) involved fixed and variable costs (Table S8). These costs were considered for the production cycle (9 months) and amortized over the same period. It was assumed that all pots in the system received an equal share of the inputs required under each substrate method, therefore, to calculate the cost of production per pot (C, KSH per pot) in each substrate method, the total cost of production (TPC, KSH per substrate method) for each system was divided by the total number of pots in each system to get average cost of production per pot. The average cost per node (C, KSH per node) was then determined using the formula C = Cost per pot/Q, where Q is the total quantity of nodes produced per pot. The continuous variable “cost per node” was then subjected to statistical analysis using SAS 9.4 version (SAS Institute Inc., 2013) to compare the cost-effectiveness of producing one node for the four genotypes in the two substrate methods using the t-test procedure. A one-way analysis-of-variance (ANOVA) model was performed and multiple comparison tests were also conducted. Further, we also used Bartlett’s test for equal variance to understand homogeneity in the variances as ANOVA assumes that the variances are homogenous. Since the distribution curve for the production cost per node may have a different shape and may not be identical, the study also used Kruskal–Wallis H test to compare the mean value of cost per node in the two substrate systems. The labor intensity could not be measured using the current experimental design due to lack of information on the amount of labor required to cultivate 1 ha of a specific crop. As a proxy for labor intensity, the study estimated the ratio of the cost of labor as a proportion of the total cost of producing a good. The higher the ratio, the higher the labor intensity. The cost per square meter was also estimated by dividing total costs by area size to understand the efficiency of resource use per square meter. The screenhouse total area was 32 m^2^ and each substrate method used half of the area with 80 observations (pots) for each substrate method.

## Results

The Analysis of Variance (ANOVA) in Table 1 shows that the variation attributable to the substrate method (sandponics system and conventional soil substrate) was significant (p < .0001) for the following vine yield characteristics: VMR, number of cuttings per vine and biomass. Morphological traits of petiole length and the vine length were not significantly different (Table 1) and therefore are not discussed. However, the substrate method significantly affected vine internode length (p ≤ 0.01) and leaf area (p ≤ 0.05) (Table 1). Table 2 shows that the means of vine internode length and leaf area were 2.5/2.7 cm and 53.4/56.1 cm^2^ for the sandponics system and conventional soil substrate method, respectively, in which the conventional soil substrate produced significantly increased growth in the vine internode and leaf area compared to the sandponics system. There was also a significant increase in the means of VMR, the number of cuttings per vine and biomass in the sandponics system (204.2; 13.6 and 121.6 g) compared to the conventional soil substrate method (163.7; 11.2 and 90.0 g) (Table 2).

**Table 1.** F values of sweetpotato vine morphological and yield characteristics of four genotypes produced under sandponics system and conventional soil substrate at KEPHIS-PQBS, Muguga, Kenya (2018–2019).

Characteristic	Substrate type	Genotype	Genotype × Substrate type interaction
Morphological			
Vine Internode length (cm)	9.22**	62.06***	2.35^ns^
Leaf area (cm^2^)	5.68*	31.95***	0.27^ns^
Petiole length (cm)	0.28^ns^	18.79***	1.74^ns^
Vine length (cm)	1.82^ns^	56.26***	0.66^ns^
Yield			
Number of plants harvested per pot	0.69^ns^	38.36***	5.01**
Vine multiplication rate per pot	119.54***	167.04***	6.42**
Number of cuttings per vine	36.77***	5.45**	6.89**
Vine weight per pot (g)	410.9***	8.85***	2.21^ns^

*: significant at p ≤ 0.05 level

**: significant at p ≤ 0.01 level

***: significant at p < .0001

ns: non-significant

**Table 2.** Means of morphological and yield characteristics of vines for four sweetpotato genotypes produced under sandponics system and conventional soil substrate at KEPHIS-PQBS, Muguga, Kenya (2018–2019).

Morphological traits	Substrate type	Genotype				Mean*
		Irene	Ejumula	Kabode	Gweri	
Vine internode length (cm)	Sandponics system	3.1	2.6	2.1	2.1	2.5B
	Conventional soil substrate	3.5	2.6	2.4	2.2	2.7A
	Mean*	3.3A	2.6B	2.2C	2.1C	Mean*
Leaf area (cm^2^)	Sandponics system	47.1	49.9	53.8	62.8	53.4B
	Conventional soil substrate	51.5	52.0	56.3	64.7	56.1A
	Mean*	49.3C	50.9C	55.1B	63.7A	
Petiole length (cm)	Mean*	10.7C	12.9A	11.9B	12.6A	
Vine length (cm)	Mean*	25.3A	20.2B	17.8C	16.9C	
Vine yield traits						
Number of plants harvested	Mean*	20.4A	16.8B	12.7C	12.2C	
Vine multiplication rate	Sandponics system	282.2	206.5	172.4	155.6	204.2A
	Conventional soil substrate	222.2	155.6	139.4	137.6	163.7B
	Mean*	252.2A	181.1B	155.9C	146.6C	
Number of cuttings per vine	Sandponics system	13.7	12.1	13.1	15.5	13.6A
	Conventional soil substrate	12.0	9.9	12.7	10.2	11.2B
	Mean*	12.8A	11.0B	12.9A	12.9A	
Biomass (g)	Sandponics system	127.0	115.9	125.2	118.3	121.6A
	Conventional soil substrate	90.2	82.9	94.4	92.6	90.0B
	Mean*	108.6A	99.4B	109.8A	105.5A	

*: different letters indicate means differed at the p ≤ 0.05 level

The ANOVA also indicates that the genotypes varied significantly (p < .0001) for vine internode length, leaf area, petiole length, and vine length but the significant variations in the vine internode length between the two substrate methods (Table 3) were only exhibited by genotypes Irene and Kabode (p = 0.005 and p = 0.003, respectively). Among the four genotypes, only the genotype Irene showed significant variation in the leaf area between the two substrate methods at p = 0.02 (Table 3). In general, genotype Irene recorded the highest mean for vine internode length (3.3 cm) and vine length (25.3 cm) compared to the other three genotypes (Table 2). Genotype Gweri had the highest mean for the leaf area (63.7 cm^2^) while genotype Irene recorded the lowest leaf area (49.3 cm^2^). Genotypes Ejumula and Gweri had the highest means for petiole length which were 12.9 cm and 12.6 cm, in that order (Table 2). Significant variations were also observed between genotypes for vine yield traits such as number of vines harvested, VMR, number of three-node cuttings per vine, and biomass at p < .0001 (Table 1). Considering vine yield traits (Table 2), genotype Irene had the highest mean VMR (252.2) for the 9-month period, number of vines harvested (20.4 vines per pot) followed by genotypes Kabode and Gweri which recorded 12.7 and 12.2 vines per pot, respectively. Genotypes Gweri, Kabode, and Irene recorded the highest means for a number of cuttings per vine (12.9; 12.9 and 12.8, in that order), subsequently, genotypes Kabode, Irene and Gweri also had the highest means for biomass (109.8 g; 108.6 g and 105.5 g, respectively).

**Table 3.** Comparing sweetpotato pre-basic seed production of four genotypes under sandponics system and conventional soil substrate using a t-test for 9-month crop calendar at KEPHIS-PQBS, Muguga, Kenya (2018–2019).

Variety yield traits	Variety	Sandponics system	Conventional soil substrate	t	p
Vine Internode length (cm)	Irene	3.1 ± 0.2	3.5 ± 0.2	−3.0	0.005
	Kabode	2.0 ± 0.1	2.4 ± 0.2	−3.4	0.003
	Ejumula	2.6 ± 0.2	2.6 ± 0.2	−0.2	0.9
	Gweri	2.1 ± 0.2	2.2 ± 0.2	−0.2	0.9
Leaf area (cm^2^)	Irene	47.1 ± 2.3	51.5 ± 2.8	−2.5	0.02
	Kabode	53.8 ± 3.4	56.3 ± 2.9	−1.1	0.3
	Ejumula	49.9 ± 2.9	52.0 ± 2.8	−1.0	0.3
	Gweri	62.8 ± 3.4	64.7 ± 4.7	−0.6	0.5
Vine multiplication rate	Irene	282.2 ± 19.5	222.2 ± 12.4	5.2	<.0001
	Kabode	172.4 ± 8.2	139.4 ± 7.8	5.8	<.0001
	Ejumula	206.5 ± 8.0	155.6 ± 7.5	9.3	<.0001
	Gweri	155.6 ± 7.0	137.6 ± 6.6	3.7	0.0006
Number of cuttings per vine	Irene	13.7 ± 1.2	12.0 ± 1.0	2.2	0.03
	Kabode	13.1 ± 1.4	12.7 ± 1.4	0.4	0.7
	Ejumula	12.1 ± 0.7	9.9 ± 0.8	4.0	0.0003
	Gweri	15.5 ± 1.4	10.2 ± 0.7	6.7	<.0001
Vine weight per pot/g	Irene	127.0 ± 4.1	90.2 ± 6.4	9.7	<.0001
	Kabode	125.2 ± 4.3	94.4 ± 3.8	10.7	<.0001
	Ejumula	115.9 ± 4.5	82.9 ± 3.9	11.0	<.0001
	Gweri	118.3 ± 3.2	92.6 ± 4.4	9.5	<.0001

The interaction between genotype and substrate type was significant for vine yield traits for example number of vines harvested (p ≤ 0.01), VMR (p < .0001) and number of cuttings per vine (p ≤ 0.01). A detailed comparison of sweetpotato vine morphological and yield traits for the four genotypes in sandponics system and conventional soil substrate using a t-test is shown in Table 3. In general, the increase in VMR, number of cuttings per vine and vine weight per pot was significant (p < 0.0001) for all the four genotypes in the sandponics system when compared to the conventional soil substrate type (Table 3).

The ANOVA (Table S10) further showed that: the effect of ratooning; interaction of variety and ratooning; and combination of substrate type and ratooning on VMR were significant at p < .0001. Moreover, the effect of interaction between variety × substrate type × ratooning on VMR was also significant at p = 0.05. Pairwise comparison by Dunnett test of the five ratoons (84, 126, 168, 210, and 252 days) after planting (DAP) compared to the first harvest (42 DAP) as the control showed that the highest significant difference was between 252 DAP and 42 DAP (Table S11) indicating that there was significant increase in VMR in the later stages of vine propagation when using ratooning technique. Also, results showed that the interaction of sandponics system × ratooning resulted to significantly increased VMR by 21.8% compared to the conventional system (Table 4 and Table S10). Among the four genotypes, Irene was the most advantageous genotype for ratooning while Kabode the least (Table S12).

**Table 4.** Comparison of sweetpotato pre-basic seed production under sandponics system and conventional soil substrate over six harvests at KEPHIS-PQBS, Muguga, Kenya in 2018–2019.

Days after planting	Vine multiplication rate		t	p
	Sandponics system	Conventional soil substrate method		
42	27.6 ± 1.2	28.3 ± 1.3	−0.8	0.4
84	28.2 ± 1.6	20.7 ± 1.2	7.5	<.0001
126	35.9 ± 2.4	25.7 ± 1.7	6.9	<.0001
168	34.8 ± 2.6	22.4 ± 1.8	7.8	<.0001
210	36.3 ± 3.1	31.6 ± 2.3	2.4	0.02
252	41.4 ± 3.3	35.1 ± 2.4	3.1	0.002
Average	**34.0 ± 1.1**	**27.3 ± 0.9**	**9.6**	**<.0001**

The findings on nutrient analysis of leaf samples from plants growing under both the sandponics system and the conventional soil substrate showed that the mean nutrient concentration for five elements (N, P, Ca, S, and B) was within the recommended range for vine propagation; but that the concentrations were significantly higher (p ≤ 0.05) for samples from the sandponics system for all the above nutrients, with the exception of Potassium and Sulfur (Table 5). Higher levels of potassium were extracted from leaves sampled from conventional soil substrate production method.

**Table 5.** Means of sweetpotato leaf tissue nutrient analysis for composite leaf samples from the sandponics system and conventional soil substrate during pre-basic seed production at KEPHIS-PQBS, Muguga, Kenya (2018–2019).

Vine Production System	Mean leaf tissue nutrient concentration					
	N (%)	P (%)	K (%)	Ca (%)	S (%)	B (ppm)
Sandponics system	4.9A	0.6A	0.7B	2.4A	0.3A	82.3A
Conventional soil substrate method	4.1B	0.4B	3.7A	1.7B	0.3A	50.7B

Means followed by different letters within a column are significantly different at 5% probability level.

The cost-effectiveness analysis results (Table 6) indicate that the cost per node produced from sandponics system is 3.5 KSH (US$ 0.035) as compared to 4.4 KSH (US$ 0.04) per node produced from conventional soil substrate method. Therefore, the cost of producing one sweetpotato node in sandponics was significantly (p < .0001) lower by 0.9 KSH (US$ 0.009), (22.7%) compared to conventional soil substrate system. To validate these results, the study also conducted regression analysis (Table S13) to understand the causation of each substrate method on cost per node by genotype. The results also validated one-way ANOVA results with strong causation effects and overall fitness of the estimated models through R^2^. The study conducted Bartlett’s test and the results were insignificant which means we cannot reject the assumption that the variances are homogenous and hence data were normally distributed.

**Table 6.** Average cost (KSH) of producing one sweetpotato node at KEPHIS-PQBS, Muguga, Kenya (2018–2019) in sandponics system compared with conventional soil substrate after six harvests for four genotypes and test of equality using one-way test.

Genotype	Sandponics system	Conventional soil substrate method	Difference	p value	Bartlett’s test	Kruskal-Wallis equality-of-populations rank test
Irene	2.449	3.141	−0.692	<.0001**	0.845	-
Kabode	3.949	4.997	−1.048	<.0001**	0.299	-
Ejumula	3.284	4.465	−1.181	<.0001**	0.020**	0.0001**
Gweri	4.37	5.049	−0.679	0.0002**	0.175	-
Overall (all genotypes)	**3.513**	**4.413**	**−0.9**	<.0001**	**0.331**	**<.0001**

Exchange rate 1 US{{footpara}}nbsp;= 100 KSH in the year 2019

*, ** indicates 5% & 1% level significant, respectively

Further, the one-way analysis of variance and regression analysis were conducted within genotypes as well. The results showed that among the four genotypes, Ejumula was the most cost-effective to produce in the sandponics system compared to the other three genotypes. The cost of producing one node of Ejumula in sandponics system was significantly (p < .0001) lower by 1.2 KSH (US$ 0.012) compared to the conventional soil substrate method. Therefore, for each node of Ejumula produced in the sandponics system, 1.2 KSH (US$ 0.012) is saved compared to the conventional soil substrate method. The Bartlett’s test for Ejumula showed that the results are significant which means we reject the assumption that the variance are homogeneous and hence data were not normally distributed. We then conducted nonparametric tests (by Kruskal–Wallis H test using STATA 14.1 version) to determine whether this statistical difference is true or not. The ANOVA results showed that the statistical differences between the two distinct substrate methods are true. A similar approach was also carried for the other three genotypes. The Bartlett’s test was not significant and hence the Kruskal–Wallis H test was not required and interpretation could be done using the one-way test directly.

In sum, sandponics is cost-effective compared to the conventional soil substrate method but the use of sandponics system should be validated for specific genotypes. For example, in our case, it is more advantageous to use the sandponics system to produce pre-basic seed for genotype Ejumula. Genotype Irene was found to have the lowest production cost but the difference in cost per node between the sandponics system and the conventional soil substrate was highest for the genotype Ejumula (i.e., 1.2 KSH or US$ 0.012). Therefore, use of sandponics system was cost-effective for the case of genotype Ejumula as compared to other three genotypes and least cost-effective for genotype Gweri for which there was a significant (p < .0001) reduction in production cost but only of 0.7 KSH (US$ 0.007)^1^ per node compared to the conventional soil substrate type. Considering labor intensity, sandponics system is more labor intensive than using the conventional soil substrate (i.e., labor intensity ratio in terms of cost ratio was 0.023 for sandponics system and 0.017 for conventional soil substrate)^1^Labor intensity (LI) defined as the amount of labor needed in production process and is calculated as the number of workers required to cultivate 1 ha of a specific crop" (Nolte and Ostermeier, 2017) pp. 432 It is used to anlayze statistical differences between two groups in a sample.

## Discussion

The highly significant (p < .0001) 21.8% increase in the VMR under the sandponics system compared to the conventional soil substrate could be attributed to more efficient use of fertilizers and water throughout the vine propagation period. This is also reflected in the findings from the leaf tissue nutrient analysis (Table 5) which showed that the leaf tissues had levels of five elements (N, P, Ca, S, and B) within the recommended range (O’Sullivan, Asher, and Blamey 1997) but that the leaf samples produced from the sandponics system had significantly higher levels. The significantly higher increase in yield could be attributed to uninterrupted and optimal nutrient and water supply in sandponics system (Sardare 2015; Wahome et al. 2011). This could also be the reason why the sandponics system significantly (p < .0001) outperformed the conventional soil substrate by 31.6% considering biomass accumulation. Our findings corroborate previous work where sand hydroponic systems have also been used successfully in the production of high-quality pre-basic seed potato (Mbiri et al. 2015; Mateus-Rodriguez et al. 2013; Tessema and Dagne 2018). Similarly, soil-less production systems for different crops have shown several benefits compared to conventional production systems (Wahome et al. 2011). One of the chief merits is that sand hydroponics system produces higher yields (Tessema and Dagne 2018).

Our findings are relevant to work conducted on potassium nutrition of sweetpotato (Byju and George 2005). The deliberate omission of potassium (K) in the optimized sweetpotato sandponics media seems to have led to an increased number of nodes based on the significant reduction in vine internode length for genotypes Irene and Kabode. The vine internode length for genotypes Irene and Kabode in the sandponics system was 3.1 cm and 2.0 cm, respectively, compared to the vines grown in the conventional soil substrate which recorded 3.5 cm and 2.4 cm, respectively. This could be attributed to high levels of K (0.21%) in the conventional soil substrate. This was later reflected in the 3% significantly higher levels of K extracted from leaf samples grown in the conventional soil substrate compared to leaf samples sourced from the sandponics system. The high K-levels contributed to the increased growth in the vine internode length as well as leaf area size at the expense of number of nodes produced translating into lower VMR in the conventional soil substrate vine production method. The early stage of vine propagation is reported to be less K-demanding than the storage root production stage in mature crops (Taraken et al. 2010). Other studies have also shown that the effect of K on the growth of sweetpotato occurred at least 7 weeks after planting (Bourke 1985; Byju and George 2005). However, our present data indicate that sweetpotato genotypes differ in K-demand during the early stages of vine propagation as exhibited by genotypes Irene and Kabode which had increased significant growth of vine internode length and leaf area in favor of the conventional soil substrate method in which the soil substrate had higher levels of K compared to the sandponics system . These results corroborate with Byju and George (2005) and Ila’ava (1997) who reported that sweetpotato cultivars differ in their tolerance to low levels of nutrients, indicating that early stages of growth for genotypes Ejumula and Gweri are less K-demanding. These findings further indicated that the growth morphology of sweetpotato genotypes seems to also play key roles in dictating the amount of K uptake, given that genotypes Irene and Kabode exhibit erect and semi-erect growth habit, respectively, large amount of K is needed by these genotypes to build cellulose, increase stalk strength and reduce lodging one of the major roles played by K (Byju and George 2005). Reduced vine internode length “rosetting” is also often associated with K deficiency (Gerardeaux et al. 2010; Hasanuzzaman et al. 2018).

The significant differences in the growth of vine and petiole length were influenced by genotypes as described by (Huamán 1991) in his studies on descriptors of sweetpotato. However, the means in the vine and petiole length could not be a true reflection given that Huaman’s measurements were on field-grown sweetpotato crop. In the present study genotypes Kabode and Gweri had significantly lower means for the vine internode length recorded as 2.2 cm and 2.1 cm, respectively, compared to 3.3 cm for genotype Irene. However, considering the number of nodes produced per vine, the means for genotypes Irene (12.8), Kabode (12.9) and Gweri (12.9) were not significantly different. These data showed that reduction in vine internode length increases VMR and this is a varietal characteristic. Our data further showed that above-ground biomass accumulation is also genotype dependent, agreeing with Bhagasari et al. (1990) findings.

The mean VMR was 33.6% higher (p ≤ 0.05) between the first harvest (42 DAP) and last (252 DAP) harvest. This could be attributed to regrowth of vines from multiple regenerating sprouts following subsequent harvests. Ratoon cropping technique in hydroponically grown pepper (*Capsicum annuum* L.) showed increased marketable fruit yields (Riga 2013). The increased fruit yields were due to a greater number of stems, 4 to 6 new re-grow stems per plant compared to newly planted plants having from 2 to 3 stems above the first fork. In this study, the 21.8% increase in VMR rates based on the interaction of the sandponics system and ratooning could be as a result of consistent supply of optimal nutrient to the multiple regenerating sprouts throughout the growing season. Ratooning technique has also been used in the production of Amaranthus (*Amaranthus cruentus* L.) which showed the total numbers of leaves and branches of Amaranthus developed was greater, gaining a higher total fresh weight yield, and the total dry weight of various plant parts and resulting in more profit at the optimum commercial stage (Fu 2008). Our results gave similar findings.

The increased VMR for the four genotypes was controlled by the increased numbers of stems (stem density) in the subsequent ratoons. For instance, each pot was planted with 10 cuttings and by the 6th harvest the stem density had significantly increased and was recorded as 20.4, 16.8, 12.7 and 12.2 for genotypes Irene, Ejumula, Kabode, and Gweri respectively. However, our data further showed that sprouting ability is genotype dependent, agreeing with Tumwegamire et al. (2014). The increased regrowth translated into an increase in stem density and subsequently, VMR in the later ratoons. There seems to be a positive correlation between ratooning and VMR which has implications for the production cost of seed with subsequent ratoons. The increased VMR reduces the unit production cost and thereby possible options for increasing profit margin. This helps seed producers to develop pricing strategies using different price points for different types of customers which will attract more clients and increase revenue (Rajendran, Kimenye, and McEwan 2017). However, the current recommended practice for sweetpotato pre-basic seed production using conventional soil substrate in screenhouses is to only ratoon three times due to concerns about the seed quality. Greater benefits will be realized from using later ratoons. Therefore, subsequent studies should investigate the effect ratooning on seed quality under sandponics system multiplication.

Turning to the analysis of the cost-effectiveness of the sandponics system and the conventional soil substrate, the significant reduction of 0.009 US$ (22.7%) in the unit production cost in the sandponics system was due to the increased VMR which was 21.8% higher compared to the conventional soil substrate although only for selected genotypes. The highly significant (p < .0001) reduction in the cost of producing one node of genotype Ejumula by US$ 0.012 which was the most cost-effective genotype to produce in sandponics is attributed to its increased VMR in sandponics compared to the conventional soil substrate. These results indicate that sandponics technology is cost-effective compared with to the conventional soil substrate for selected genotypes. The increased labor intensity in sandponics is attributed to more labor time required to measure precisely the optimal soluble inorganic fertilizers during nutrient media preparation. However, with the optimization of a nutrient media for sweetpotato vine multiplication in sandponics (Makokha et al. 2018) development of a pre-mixed soluble inorganic fertilizer will reduce the labor and increase the efficiency of sandponics technology. Although our results have indicated that sandponics technology is cost-effective compared to the use of conventional soil substrate, sandponics system can be more effective for specific genotypes rather than all genotypes. The reasons for this include (i) the current nutrient media mix is more suited for some genotypes, for example, in our case Ejumula, (ii) purchase, transport and sterilization of sand is cheaper (0.035 US$ Kg^−1^) since it was readily available and the cost involved in sterilizing is low compared to the soil substrate (0.2 US$ Kg^−1^) cost, (ii) increased VMR in the sandponics compared to the conventional system.

In the principle of economics, a change in technology in the production system will have an impact on demand and supply which influences prices in the market (Gittinger 1985). For example, when a new technology is discovered in a production system and allows the system to produce at lower costs, this will lead to a larger quantity to be produced at a lower price which will increase the economies of scale and overall revenue will also increase. Thus, sandponics system (new technology) increases the pre-basic seed production efficiency attributed to an increased VMR compared to the use of conventional soil substrate and provides a lower price to end-users. Research has shown that if pre-basic seed is cheaper (Mbiri et al. 2015) then, the production cost for the following certified generations will also be cheaper.

The major impediment to using sandponics system is that soluble inorganic fertilizers are not readily available in some areas of SSA countries, therefore, further studies are required to explore alternative sources of nutrients that are locally available, for example, manure and compost filtrates.

## Conclusions and recommendations

This study found out that sandponics system is a cost-effective technology compared to the use of a conventional soil substrate. However, sandponics system is more effective for selected genotypes and hence it is necessary to identify the appropriate genotypes for mass multiplication using sandponics system. In this experiment, although the production cost per node was lowest for genotype Irene, the difference in costs between the two methods was higher for genotype Ejumula and hence it was found to be the most cost-effective genotype of the four tested to use in sandponics system. Other results (Makokha, P., Ssali, R.T., Wanjala, B.W., Rajendran, S., McEwan, M.A., and Low, W.J. 2019, unpublished) on the root yield potential of sweetpotato vines produced using sandponics system showed a significant increase in vine survival, storage root yield, number of roots per plant and fresh foliage yield compared to vines sourced from the conventional soil substrate. Sandponics system is a feasible alternative technology for pre-basic sweetpotato seed production. However, more studies are required on: (i) evaluating the vigor and quality of subsequent basic seed production using starter materials sourced from the sandponics system, (ii) assessing the maximum number of ratoons that can be reached taking into account both quality as planting material and timing of seed availability in relation to market demand, (iii) evaluating planting in benches and troughs vs. pots to optimize on plant density, (iv) effect of ratooning on vine quality and (v) adapting the sandponics system for high throughput phenotyping platforms or assays for some traits that are either difficult to measure under field conditions or are likely to be suitable for cropping models accounting for the effects of climate change. These could include traits like moisture stress resistance, nutrient use efficiency, cold stress tolerance or heat stress tolerance, viral and fungal bio-assays in sweetpotato among others.

## Supplementary material

The supplemental data for this article can be accessed here.
