# Supplementary material for: Comparative analysis for producing sweetpotato pre-basic seed using sandponics and conventional systems
Source: J Crop Improv. 2019 Oct 12;34(1):84–102. doi: 10.1080/15427528.2019.1674758 (PMC7363038; doi:10.1080/15427528.2019.1674758)
Supplement: Supplemental Material [file WCIM_A_1674758_SM3824.docx]

**Supplementary Table 1**: Chemical and physical properties of sterilized soil substrate sampled from screenhouse pots at KEPHIS-PQBS, Muguga, Kenya prior to planting of sweetpotato pre-basic seed using conventional soil substrate method (July 2018).

| Parameter | Unit | Result | Description* |
| --- | --- | --- | --- |
| Sampling depth | m | 0.3 |  |
| pH (H_2_O) |  | 7.52 | High |
| EC (salts) | uS/cm | 1250 | High |
| Phosphorus (Olsen) | **(ppm)** | **28.6** | **Low** |
| Potassium | **ppm** | **2060** | **Low** |
| Calcium | **ppm** | **3780** | **Low** |
| Magnesium | ppm | 547 | Low |
| Sulfur | **ppm** | **106** | **Low** |
| Iron | ppm | 77.5 | Optimum |
| Manganese | ppm | 318 | Optimum |
| Boron | **ppm** | **2.16** | **Low** |
| Copper | ppm | 1.35 | Low |
| Zinc | ppm | 16.5 | Optimum |
| C.E.C | Mq/100q | 31.4 | High |
| Total N | **%** | **0.21** | **Low** |
| Organic matter | % | 5.83 | Optimum |
| C/N ratio |  | 16.1 | Optimum |

**Recommendations based on O’Sullivan et al. (1997).*

**Supplementary Table 2:** Water source from KEPHIS-PQBS, Muguga, Kenya, its nutrient composition and adjustments for preparation of nutrient media for sweetpotato pre-basic seed multiplication using sandponics system (2018 – 2019).

|  | **N (ppm)** | **P (ppm)** | **Ca (ppm)** | **S (ppm)** | **B (ppm)** |
| --- | --- | --- | --- | --- | --- |
| Water | 0.91 | 0.059 | 4.29 | 1.86 | <0.01 |
| Makokha et al., 2018 | 200 | 60 | 200 | 120 | 0.3 |
| Adjustment | 199.1 | 59.9 | 195.71 | 118.1 | - |

**Supplementary Table 3:** Workings for nutrient media concentrations for sweetpotato pre-basic seed multiplication using sandponics system at KEPHIS-PQBS, Muguga, Kenya (2018 – 2019).

| Element | Source | Concentration and weight (g) in 1000 L |
| --- | --- | --- |
| Calcium | Calcium nitrate (15.5%N, 19%Ca) | 195.71 Ca = 1030.05 g of Ca (NO_3_)_2_ which also comes with 159.66 ppm N leaving N still needed = 199.1 less 159.66 = 39.44 ppm |
| Nitrogen | Magnesium nitrate 10%Mg, 10%N) and Calcium nitrate | 39.44 ppm of N = 394.3 g of Mg (NO3)_2_ |
| Phosphorus | Calcium triple super phosphate (46%P_2_O_5_) | 59.9 ppm of P = 298.5 g of TSP |
| Sulfur | Magnesium sulfate (26.3%S) | 118.1 ppm = 449.2 g of MgSO_4_ |
| Boron | Microsol B (2.5%B) | Microsol B (2.5%B) |

| **Supplementary Table** 4: Input cost template micro-log sheet for sweetpotato pre-basic seed multiplication using sandponics system and conventional soil substrate method at KEPHIS-PQBS, Muguga, Kenya (2018 – 2019). | | | | | | | | | | | | | |
| --- | --- | --- | --- | --- | --- | --- | --- | --- | --- | --- | --- | --- | --- |
| S. No | **Date of use** | **Particulars (Items)** | **Experiment Name for used inputs (1=Sandponics system; 2=conventional soil substrate method)** | **1=Input; 2=Consumable good; 3=Service Costs; 4=Other costs** | **Quantity purchased** | **Unit Name for QTY purchased** | **Price per unit** | **Total Price paid** | **Currency Name** | **Qty**  **Used** | **Unit Name for Qty used** | **Qty contained per purchased unit** | **Status of usage (1=Ongoing; 2=Completed)** |
| 1 |  |  |  |  |  |  |  |  |  |  |  |  |  |
| 2 |  |  |  |  |  |  |  |  |  |  |  |  |  |

**Supplementary Table 5:** Labor cost template micro-log sheet for sweetpotato pre-basic seed multiplication in sandponics system and conventional soil substrate method at KEPHIS-PQBS, Muguga, Kenya (2018 – 2019).

| LABOUR COST | |  |  |  |  |  |
| --- | --- | --- | --- | --- | --- | --- |
| NAME OF THE STAFF | |  |  |  |  |  |
| LOG SHEET FILLED BY FIELD STAFF | | | | | | |
| S.No | **Activity (mentioned type of the input product used)** | **Work involved in the type of experiment (1=Sandponics system; 2=Conventional soil substrate method)** | **Date of application (dd/mm/yy)** | **Starting Time of application (HH/MM)** | **Ending time of application (HH/MM)** | **Status of usage (1=ongoing; 2=completed)** |
| 1 |  |  |  |  |  |  |

**Supplementary Table 6:** Input template macro-log sheet for sweetpotato pre-basic seed multiplication using sandponics system and conventional soil substrate method at KEPHIS-PQBS, Muguga, Kenya (2018 – 2019).

|  | Cost Summary | | | | | | | | |  | |  | |  | |  | |  | |  | |  | |  | |  | |  | |
| --- | --- | --- | --- | --- | --- | --- | --- | --- | --- | --- | --- | --- | --- | --- | --- | --- | --- | --- | --- | --- | --- | --- | --- | --- | --- | --- | --- | --- | --- |
| S.No | **APPLICATION DATE** | **Particulars (Items)** | **Quantity purchased** | **Unit Name for QTY purchased** | **Total Price paid** | **Currency Name** | **Quantity used** | **Unit Name for Qty used** | **conversion** | | **local conversion unit** | | **Per unit cost used** | | **Total Cost for used qty** | | **Exchange Rate (1 USD = in Local Currency) during the purchase time** | | **Total Cost (in USD)** | | **conventional soil substrate method ratio for sandponics system** | | **Total Cost of qty used for coventional soil substrate method** | | **conventional ratio for CONVENTIONAL SOIL SUBSTRATE METHOD** | | **Total Cost of qty used for coventional soil substrate method** | |  |
| 1 |  |  |  |  |  |  |  |  |  | |  | |  | |  | |  | |  | |  | |  | |  | |  | |  |

**Supplementary Table 7:** Labor cost macro-log sheet for sweetpotato pre-basic seed multiplication using sandponics system and conventional soil substrate method at KEPHIS-PQBS, Muguga, Kenya (2018 – 2019).

|  |  |  |  |  |  |  |  |  |  |  |
| --- | --- | --- | --- | --- | --- | --- | --- | --- | --- | --- |
|  | **Figures** | **Unit name** |  |  |  |  |  |  |  |  |
| No of hours per day |  | hours |  |  |  |  |  |  |  |  |
| wage rate per day |  | DAY |  |  |  |  |  |  |  |  |
| Total Area Measurement |  | SQUARE METER |  |  |  |  |  |  |  |  |
|  |  |  |  | **sweetpotato vine** |  |  |  |  |  |  |
| Name of the laborer | **Starting Date (DD/MM/YYYY)** | **Ending Date (DD/MM/YYYY)** | **Wage Rate in local currency per day** | **Total Hours worked during production cycle** | **Total** | **Estimated cost (Local Currency)** | **If there is a contract, then Contract labour cost** | **Estimated total cost (Local Currency)** | **Average Exchange rate during the production period** | **Total Cost in USD** |
|  |  |  |  |  | **man-days** |  |  |  |  |  |
|  |  |  |  |  |  |  |  |  |  |  |

**Supplementary Table 8:** Detailed cost of production for sweetpotato pre-basic seed multiplication using sandponics system and conventional soil substrate method at KEPHIS-PQBS, Muguga, Kenya (2018 – 2019).

| Cultivation technique | Sandponics system |  |  |  | |
| --- | --- | --- | --- | --- | --- |
| Structure under production | Screenhouse |  |  | | |
| Local currency | KSH | Kenyan shillings |  |  | |
|  | Size | Unit name |  |  | |
| Total area measurement (m^2^) | 16 | Square meters (SQM) |  | | |
|  | Name of the unit | Measure |  |  | |
| Production unit | NODE | 1 |  |  | |
|  | FROM | TO | Production Cycle | Units | |
| Year of Calculation | June-2018 | March-2019 | 9 | Months | |
| Varieties Name | Kabode, Irene, Ejumula, Gweri | | | | |
| Number of Varieties | 4 | | | | |
| Number of harvesting | 6 | | | | |
| Irrigation / Fertigation method | Drip | | | | |
|  | | | | | |
| Yield | YIELD FOR SANDPONICS SYSTEM | | YIELD FOR CONVENTIONAL SOIL SUBSTRATE METHOD | | |
|  | NODE |  | NODE |  | |
|  | 48996 | 16 SQM | 39293 | 16 SQM | |
| PRODUCTION – SANDPONICS SYSTEM | | | | | |
| CROP NAME |  | Name of the Unit | Actual Quantity Produced | Wastages during production period (production unit) | |
| Sweetpotato vines (pre-basic) |  | Node | 48996 | 2449.8 | |
| PRODUCTION – CONVENTIONAL SOIL SUBSTRATE METHOD | | | | | |
| CROP NAME |  | Name of the Unit | Actual Quantity Produced | Wastages during production period (production unit) | |
| Sweetpotato vines |  | Node | 39293 | 1964.65 | |
| INPUT COSTS FOR THE PRODUCTION | | | | | |
| **Variable Costs** | | | | | |
| **Labor costs** | Unit | Unit Name | Total Man-days | SANDPONICS SYSTEM | CONVENTIONAL SOIL SUBSTRATE METHOD |
| Total Labor cost incurred – CONVENTIONAL |  |  | 4.9 |  | 2823 |
| DAILY OR CASUAL LABOR COST – SANDPONICS |  |  | 6.6 | 3799 |  |
| **Total** |  |  | **11.5** | **3799** | **2823** |
| Variable costs |  | | | SANDPONICS SYSTEM | CONVENTIONAL SOIL SUBSTRATE METHOD |
| Input Cost^a^ |  |  |  |  |  |
| Chemicals / Fertilizers |  |  |  | 11961 | 16113 |
| Consumables |  |  |  | 550 | 550 |
| **Total Input Costs** |  |  |  | 12511 | 16663 |
| **Total Variable Costs** |  |  |  | **16310** | **19486** |
| **Fixed Costs (Ownership costs or Capital costs)** |  |  |  | SANDPONICS SYSTEM | CONVENTIONAL SOIL SUBSTRATE METHOD |
| Screen house |  |  |  | 68,174 | 68,174 |
| Water tank and pipe, drip, stand |  |  |  | 67,018 | 67,018 |
| Pots / plates |  |  |  | 2,185 | 2,185 |
| Wheel barrow |  |  |  | 0.5 | 0.5 |
| Spade |  |  |  | 0.1 | 0.1 |
| Sprayer |  |  |  | 10.5 | 10.5 |
| Secateurs |  |  |  | 2.2 | 2.2 |
| Weighing scale |  |  |  | 32 | 0 |
|  | | | | | |
| Total Fixed Costs |  |  |  | 137,421 | 137,390 |
| Total Costs | Total variable costs + total fixed costs | | | **153,731** | **156,875** |
| Overhead Cost (water, electricity, maintenance, security and land) | 10% | | | 154 | 157 |
| Total Costs | Include overhead and contingencies & exclude wastages | | | **153,885** | **157,032** |
| Unit Cost | Total costs / total quantity of Production (excluding wastage cost) | | | **3.1** | **4.0** |
| Total costs for wastage | Unit costs*total quantity wastage | | | **7694** | **7852** |
| Total Costs | Include overhead and contingencies & include wastages cost | | | **161,579** | **164,884** |
| BREAKEVEN COST TO TOTAL COSTS | INCLUDING WASTAGES | | | **3.3** | **4.5** |
|  | | | | | |
| **COST PER VARIETY** | KSH | | | 40395 | 41221 |
|  | | | | | |
| Ejumula | Number of nodes among all 4 blocks | | | 12388 | 9338 |
| Kabode | Number of nodes among all 4 blocks | | | 10341 | 8364 |
| Irene | Number of nodes among all 4 blocks | | | 16933 | 13333 |
| Gweri | Number of nodes among all 4 blocks | | | 9334 | 8258 |
|  | | | | | |

**Note: ‘a’ inputs includes such as triple super phosphate, magnesium nitrate, magnesium sulphate, etc.**

**Supplementary Table 9:** Cost of producing sterilized sand over 5 days’ time period for sweetpotato pre-basic seed multiplication using sandponics system at KEPHIS-PQBS, Muguga, Kenya (2018 – 2019).

| **S.NO** | **PARTICULARS** | **QUANTITY** | **LOCAL UNIT** | **COST PER UNIT (KSH)** | **TOTAL COSTS (KSH)** |
| --- | --- | --- | --- | --- | --- |
| **VARIABLE COSTS** | | | | | |
| **INPUT COSTS** |  |  |  |  |  |
|  | SAND | 480 | kgs | 1.5 | 720 |
|  | JIK | 2 | lit | 80 | 160 |
| ***Sub-total*** |  |  |  |  | 880 |
| **LABOUR COSTS** |  |  |  |  |  |
|  | Daily laborers cost | 1.00 | man-days | 575 | 576 |
| ***Sub-total*** |  |  |  |  | 576 |
| **TOTAL VARIABLE COSTS** | | | | | 1456 |
| **FIXED COST** | | | | | |
|  | Drum | 3 | drums | 2.41 | 7.2 |
|  | Spade | 3 | spade | 0.2 | 0.6 |
|  | Wheel borrow | 1 | wheel borrow | 2.0 | 2.0 |
|  | Shovel | 2 | shovel | 0.2 | 0.5 |
|  | Sieve | 1 | sieve | 2.9 | 2.9 |
| TOTAL FIXED COSTS (After depreciation) | | | | | 13.2 |
| TOTAL COST EXCL. OVERHEAD & CONTIGENCY (KSH) | | | | | 1469 |
| TOTAL COST EXCL. OVERHEAD & CONTIGENCY (USD) | | | | | 15 |
| OVERHEAD COSTS (10 %) | | | | | 147 |
| RISK COSTS (5 %) | | | | | 73 |
| TOTAL COST INCL OVERHEAD & CONTIGENCY COSTS (KSH) | | | | | 1689 |
| EXCHANGE RATE | AS ON 8 APRIL 2019' | | | | 100 |
| TOTAL COST INCL OVERHEAD & CONTIGENCY COSTS (USD) | | | | | 17 |
| TOTAL QTY PRODUCED (KG) | | | | | 480 |
| BREAK EVEN COST PER UNIT (KSH) (Total costs / Total Quantity produced) | | | | | 3.5 |
| BREAK EVEN COST PER UNIT (USD) | | | | | 0.035 |

**Note: Price of sterilized soil is 20 KSH (US$ 0.20) per Kg purchased from KEPHIS-PQBS, Muguga, Kenya (2018-2019).**

**Supplementary Table 10:** The F values for vine multiplication rate of the four sweetpotato genotypes as influenced by ratooning under sandponics system and conventional soil substrate method at KEPHIS-PQBS, Muguga, Kenya (2018 – 2019).

| Yield trait | Ratoon | Ratoon × Substrate type | Variety × Ratoon | Variety × Substrate type× Ratoon |
| --- | --- | --- | --- | --- |
| Vine multiplication rate | 95.51** | 21.07** | 10.41** | 1.69* |
| p value | <.0001 | <.0001 | <.0001 | 0.05 |

**Supplementary Table 11:** Sweetpotato pre-basic seed multiplication using sandponics system and conventional soil substrate method for the 5 ratoons when compared to the plant crop (42 DAP) as the control at KEPHIS-PQBS, Muguga, Kenya (2018 – 2019).

| Ratoon comparison to the control | Difference between means | |
| --- | --- | --- |
|  | **Number of nodes produced/pot** | **Vine multiplication rate** |
| 84-42 | -10.6* | -3.5* |
| 126-42 | 8.7* | 2.9* |
| 168-42 | 1.9 | 0.6 |
| 210-42 | 17.9* | 6.0* |
| 252-42 | 31.0* | 10.3* |

*significantly different at 5% level of confidence according to Dunnett’s test

**Supplementary Table 12**: Pairwise multiple comparison of sweetpotato vine multiplication rate due to interaction between genotype and ratooning under sandponics system and conventional soil substrate method during pre-basic seed multiplication at KEPHIS-PQBS, Muguga, Kenya (2018 – 2019).

| Comparison of harvest 6^th^ , 5^th^ , 4^th^ , 3^rd^ & 2^nd^ to the 1^st^ harvest done at 42-day intervals | VMR (difference between means) | | | |
| --- | --- | --- | --- | --- |
|  | **Irene** | **Ejumula** | **Kabode** | **Gweri** |
| 252-42 | 19.3* | 10.7* | 5.3* | 5.9* |
| 210-42 | 16.2* | 4.8* | 1.0 | 1.8 |
| 168-42 | 8.7* | -1.8 | -3.3 | -1.1 |
| 126-42 | 9.5* | 0.7 | -0.7 | 2.1 |
| 84-42 | -3.6 | -2.3 | -4.9* | -3.4 |

*Significant at 0.05 level of probability confidence according to Dunnett test.

**Supplementary Table 13:** Regression analysis on measuring causation effect of sandponics system vs conventional soil substrate method on cost per node for each genotype during sweetpotato pre-basic seed multiplication at KEPHIS-PQBS, Muguga, Kenya (2018 – 2019).

| Genotype | Ejumula | Kabode | Irene | Gweri | Overall |
| --- | --- | --- | --- | --- | --- |
| Conventional soil substrate method (Base) | - | - | - | - | - |
| Sandponics system | -1.18 (-9.24)** | -0.68 (-4.16)** | -0.69 (-5.06)** | -1.05 (-6.44)** | -0.90 (-6.45)** |
| R^2^ |  |  |  |  |  |
| *, ** indicates 5% & 1% level significant respectively; in parenthesis is t-value | | | | |  |
